# Neuroprotective Effect of Sodium Butyrate against Cerebral Ischemia/Reperfusion Injury in Mice

**DOI:** 10.1155/2015/395895

**Published:** 2015-05-07

**Authors:** Jing Sun, Fangyan Wang, Haixiao Li, Huiqing Zhang, Jiangtao Jin, Wenqian Chen, Mengqi Pang, Junjie Yu, Yiwen He, Jiaming Liu, Chunfeng Liu

**Affiliations:** ^1^Department of Neurology, The Second Affiliated Hospital of Soochow University, Suzhou, Jiangsu 215004, China; ^2^Department of Neurology, The Second Affiliated Hospital of Wenzhou Medical University, Wenzhou, Zhejiang 325027, China; ^3^School of Basic Medical Sciences, Wenzhou Medical University, Wenzhou, Zhejiang 325035, China; ^4^School of Environmental Science and Public Health, Wenzhou Medical University, Wenzhou, Zhejiang 325035, China

## Abstract

Sodium butyrate (NaB) is a dietary microbial fermentation product of fiber and serves as an important neuromodulator in the central nervous system. In this study, we further investigated that NaB attenuated cerebral ischemia/reperfusion (I/R) injury in vivo and its possible mechanisms. NaB (5, 10 mg/kg) was administered intragastrically 3 h after the onset of reperfusion in bilateral common carotid artery occlusion (BCCAO) mice. After 24 h of reperfusion, neurological deficits scores were estimated. Morphological examination was performed by electron microscopy and hematoxylin-eosin (H&E) staining. The levels of oxidative stress and inflammatory cytokines were assessed. Apoptotic neurons were measured by TUNEL; apoptosis-related protein caspase-3, Bcl-2, Bax, the phosphorylation Akt (p-Akt), and BDNF were assayed by western blot and immunohistochemistry. The results showed that 10 mg/kg NaB treatment significantly ameliorated neurological deficit and histopathology changes in cerebral I/R injury. Moreover, 10 mg/kg NaB treatment markedly restored the levels of MDA, SOD, IL-1*β*, TNF-*α*, and IL-8. 10 mg/kg NaB treatment also remarkably inhibited the apoptosis, decreasing the levels of caspase-3 and Bax and increasing the levels of Bcl-2, p-Akt, and BDNF. This study suggested that NaB exerts neuroprotective effects on cerebral I/R injury by antioxidant, anti-inflammatory, and antiapoptotic properties and BDNF-PI3K/Akt pathway is involved in antiapoptotic effect.

## 1. Introduction

Accumulating evidence has demonstrated that cerebral ischemia/reperfusion (I/R) injury often causes irreversible brain damage and the cascade of events causing neuronal injury and death, which is involved in many complex factors such as deprivation of blood flow, oxidative stress, inflammation, and apoptosis [1, 2]. The cerebral I/R injury can result in functional impairment and/or neuronal death [3]. Oxidative stress is one of the primary factors that can aggravate the cerebral I/R injury. Excessive production of reactive oxygen species (ROS) may directly contribute to destruction of the cell membrane by inducing lipid peroxidation [4]. In addition, antioxidant is an effective substance, which has been shown to alleviate neuronal damage from brain ischemia injury [5]. Furthermore, significant inflammatory response has been observed during ischemic stroke [2]. Numbers data has indicated that various inflammatory mediators such as IL-1*β*, IL-8, and TNF-*α* can lead to neuronal damage [6, 7]. Apoptosis is generally considered to play a major role in delayed neuronal death following cerebral I/R [8]. Several studies have shown that many signal pathways could be involved in antiapoptosis and phosphoinositide 3-kinase/protein kinase B (PI3K/Akt) signaling pathway is essential for the cell growth and survival in brain I/R injury [9]. Taking into account the fact that cerebral I/R injury is associated with the oxidative, inflammatory, and apoptotic mechanism, some are is willing to explore the neuroprotective agents that may attenuate the cerebral I/R damage. At present, natural products, especially microbial fermentation products, probably represent an ideal source to develop safe and effective agents for management of cerebral I/R injury.

Sodium butyrate (NaB), a dietary microbial fermentation product of fiber, is a sort of short chain fatty acids (SCFAs), which has been previously shown to inhibit intestinal pathogenic bacteria and maintain gastrointestinal homeostasis [10]. Recent studies have indicated that NaB could provide anti-inflammatory and neuroprotective effects in neurodegenerative disorders [11] and also improve spatial learning and memory ability [12], myocardial I/R injury [13], acute lung injury [14], and acute liver failure [15]. Although the neuroprotective effect of NaB has been well studied, its neuroprotective effects on the cerebral I/R injury have not yet been clear. Therefore, the present study was performed to investigate the neuroprotective effect and possible mechanisms of NaB treatment in cerebral I/R injury induced by the bilateral common carotid arteries occlusion (BCCAO) in mice.

## 2. Materials and Methods

### 2.1. Animals

All procedures involving animals were approved by and conformed to the guidelines of the Institutional Animal Care Committee of Wenzhou Medical University. Male ICR mice (6-week old, 22–24 g) were obtained from Experiment Animal Center of Wenzhou Medical University. The mice were housed in a pathogen-free animal facility and maintained on a 12 h light/dark cycle. Food and water were available* ad libitum* [16].

### 2.2. Surgical Procedures of Cerebral I/R Injury Mice Model

Cerebral I/R injury mice model was induced by the bilateral common carotid arteries occlusion (BCCAO) with vascular clips [17, 18]. The mice were anaesthetized with 400 mg/kg injection of chloral hydrate and a midline incision in the ventral side of the neck was made to expose the right and left common carotid arteries. We gently separated the two arteries and occluded them with vascular clips for 20 min, and then the clips were removed to restore blood for recirculation. The sham-operated mice underwent the same operation procedure, but the common carotid arteries were not occluded [19, 20].

### 2.3. Drug Administration and Groups

NaB (sodium butyrate, sigma Co. Ltd., USA with a purity ≥99%) was dissolved in normal saline and intragastrically administered 3 h after the onset of reperfusion in mice. The mice were randomly divided into four groups (*n* = 12) as follows: (1) sham-operated group; (2) cerebral I/R model group; (3-4) NaB treatment groups, which were subjected to I/R model and treated with NaB 5 and 10 mg/kg, respectively. Sham-operated and model groups were given an identical dose of normal saline to serve as a control.

### 2.4. Neurological Function Assessment

Evaluation of the neurological deficits at 24 h after the reperfusion was based on the method of Longa [13]. Neurological function was scored on a five-point scale: 0, no deficits; 1, difficulty in fully extending the contralateral forelimb; 2, not being able to extend the contralateral forelimb; 3, mild circling to the contralateral side; 4, severe circling; and 5, falling to the contralateral side. The higher the neurological deficit score is, the severer the injury is. Ten mice were evaluated in each group.

### 2.5. Morphological Evaluation

After 24 h of reperfusion, brain tissues were rapidly removed and fixed with 25% glutaraldehyde for 2 h at 4°C, rinsed with PBS, and soaked in osmium tetroxide. Ultrathin sections were prepared and placed onto colloid coated copper grids and double-stained with 0.4% uranyl acetate and 2% lead acetate. The ultrastructure of the neurons was observed by electron microscopy.

The brains were processed routinely for paraffin embedding and sectioning into 5 *μ*m thickness, and the sections were stained with hematoxylin-eosin (H&E) according to the methods described previously [21]. The normal tissue was stained to deep red-blue and the ischemia area was stained to pale pink. The slides were photographed under light microscope.

### 2.6. Biochemical Determination

Brain tissues were weighed and homogenized for MDA, SOD activity, IL-1*β*, IL-8, and TNF-*α* assay in conformance with ELISA kits. The content of MDA and enzymatic activities of SOD were measured using ELISA kits (Nanjing Jiancheng Bioengineering Institute), and the contents of IL-1*β*, IL-8, and TNF-*α* were measured using ELISA kits (RayBiotech, Norcross, GA, USA).

### 2.7. TUNEL Staining

Paraffin-embedded brains were sectioned in 5 *μ*m thickness and stained with TUNEL assay kit for apoptotic cells, according to the previous methods [16]. The positive cells displayed a brown stain within the cytoplasm of the apoptotic cells [22]. The slides were photographed (400x magnification).

### 2.8. Western Blotting

A total of 20 *μ*g protein samples, obtained from the ischemic brain tissues, were loaded onto a 12% SDS-PAGE gel. After the electrophoresis, proteins were transferred to a nitrocellulose membrane. Nonspecific binding sites were blocked with 5% nonfat milk in TBST for 1 h and then incubated with primary antibodies against BDNF, Akt, p-Akt, Bcl-2, Bax, caspase-3, and *β*-actin (1 : 1000, Bioworld, USA) overnight at 4°C. Subsequently, blots were incubated with HRP-conjugated secondary antibody at room temperature for 1 h. The protein bands were visualized with ECL chemiluminescence system (Thermo Scientific, Rockford, IL). The optical density was quantified by performing the National Institute of Health ImageJ software. *β*-actin was performed as a loading control.

### 2.9. Immunohistochemistry

Immunohistochemistry for Bcl-2 and Bax was carried out on paraffin-embedded sections. The primary antibody of Bcl-2 (1 : 100, Cell Signaling Technology, USA) and Bax (1 : 250, Bioworld, USA) was applied overnight at 4°C. The sections were washed and then incubated with secondary antibodies (goat-anti-rabbit, 1 : 500), and the sections were visualized using diaminobenzidine (DAB) as the chromogen. Brown granules in cells under microscope were defined as positive signals and observed at 400x magnification of light microscopy.

### 2.10. Statistical Analysis

The data were expressed as mean ± SD and analyzed using the SPSS 17.0 statistical analytical software (SPSS, Chicago, IL, USA). Statistical analysis of the results was carried out by one-way ANOVA, followed by fisher post hoc comparisons. Statistical significance was assumed for *p* < 0.05.

## 3. Results

### 3.1. NaB Improved Neurological Deficit

**Figure 1:**
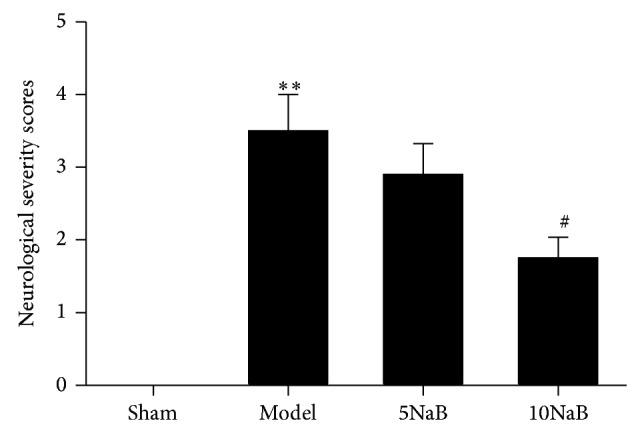
Effect of NaB on neurological deficit scores. ^∗∗^ *p* < 0.01 versus sham group and ^#^ *p* < 0.05 versus model group. *n* = 10, each group.

The ischemia of the cerebral hippocampus caused a deficit in neurological function in the mice that was mainly visible as left forelimb paralysis. In this study, we examined the neuroprotective effect of NaB treatment using neurological deficit scores. As shown in Figure 1, all mice except sham-operated ones presented neurological deficit (*p* > 0.05). The mice in 10 mg/kg NaB treatment groups exhibited significantly improved neurological deficits scores after the cerebral I/R injury compared with the model group (*p* < 0.01). These results suggested that 10 mg/kg NaB treatment effectively attenuated the neurological dysfunction of cerebral I/R in mice.

### 3.2. NaB Protects against Histological Changes

**Figure 2:**
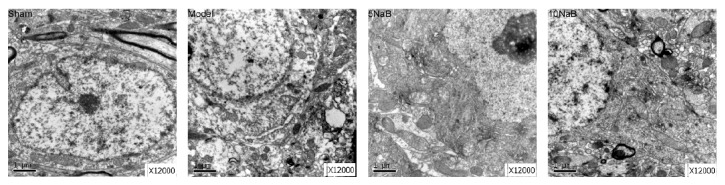
Effect of NaB on ultrastructure. Representative photomicrographs of ultrastructure; magnification: 12,000x. Scale bar = 1 *μ*m.

As shown in Figure 2, no destructive changes were observed in the sham group by electron microscopy; the normal neurons contained large oval nucleuses and clear mitochondria. The irregular nucleuses of neurons and swollen mitochondria were observed in the model mice. By contrast, NaB attenuated the impairment of the ultrastructural neurons in the cerebral I/R injury mice.

**Figure 3:**
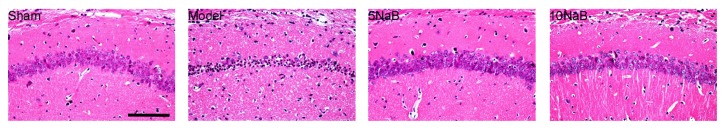
Effect of NaB on histopathology. Representative photomicrographs of H&E staining. Cells with brown stained cytoplasm are positive cells. Magnification: 400x. Scale bar = 20 *μ*m.

The histopathological abnormalities in the hippocampal CA1 region were measured by the H&E staining. As illustrated in Figure 3, shrunken and pycnotic nuclei of CA1 neurons were exhibited in the model group. By contrast, these neuronal damages were dramatically reduced by the NaB treatments.

### 3.3. NaB Attenuates the Oxidative Stress

**Figure 4:**
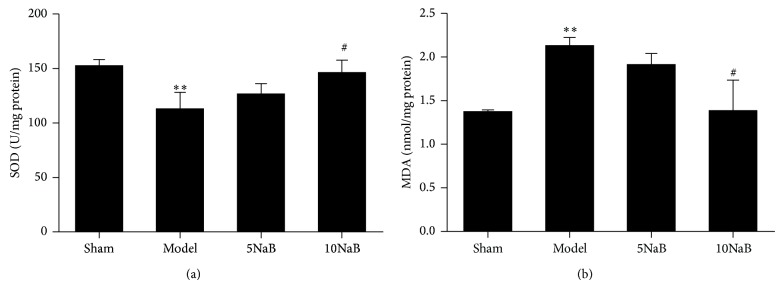
Effect of NaB on the levels of SOD and MDA. SOD assay (a) and MDA assay (b). ^∗∗^ *p* < 0.01 versus sham group and ^#^ *p* < 0.05 versus model group. *n* = 6, each group.

To investigate the effects of NaB on lipid peroxidation and free radical activity induced by BCCAO, the contents of MDA and the activities of SOD in the brain tissues were measured. After 24 h of reperfusion, the activities of SOD in ischemic hippocampus of the model group were diminished remarkably compared with the sham group (*p* < 0.01, Figure 4(a)). However, the SOD activity in the 10 mg/kg NaB treatment group was significantly enhanced compared with the model group (*p* < 0.05, Figure 4(a)). The content of MDA was also increased in the model group compared with the sham group (*p* < 0.01, Figure 4(b)). After 10 mg/kg NaB treatment, the content of MDA was evidently deceased in comparison to the model group (*p* < 0.05, Figure 4(b)). These results suggested that NaB (10 mg/kg) treatment effectively attenuated the brain oxidative stress of the cerebral I/R in mice.

### 3.4. NaB Attenuates the Inflammatory Response

**Figure 5:**
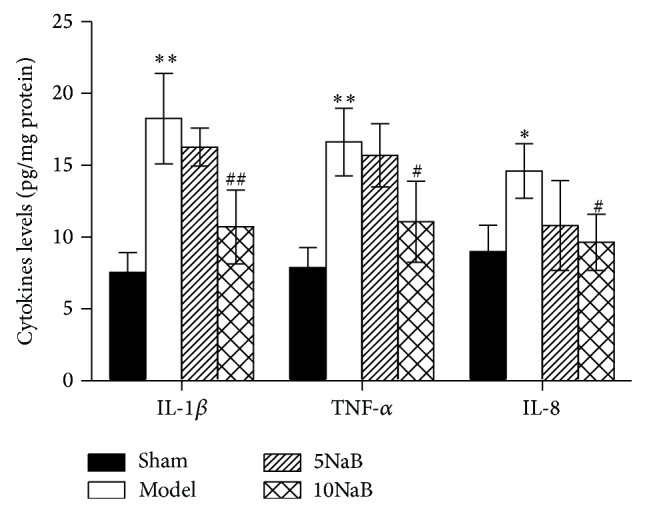
Effect of NaB on the levels of IL-1*β*, IL-8, and TNF-*α*. ^∗^ *p* < 0.05 versus sham group, ^∗∗^ *p* < 0.01 versus sham group, ^#^ *p* < 0.05 versus model group, and ^##^ *p* < 0.05 versus model group. *n* = 6, each group.

Inflammation is one of the key pathogenic events in the cerebral I/R injury. We examined the cytokines (IL-1*β*, IL-8, and TNF-*α*) in the ischemic hippocampus after 24 h of reperfusion by ELISA. As shown in Figure 5, levels of IL-1*β* (*p* < 0.01), IL-8 (*p* < 0.05), and TNF-*α* (*p* < 0.01) were markedly increased in the model group relative to the sham group. However, 10 mg/kg NaB treatment group significantly decreased the levels of these cytokines relative to the model group (*p* < 0.05), especially the level of IL-1*β* (*p* < 0.01).

### 3.5. NaB Attenuates the Apoptosis on the Hippocampus

**Figure 6:**
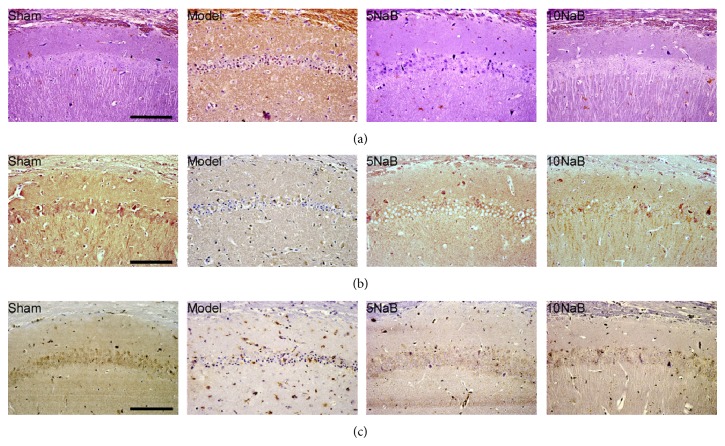
NaB attenuates the apoptosis on the hippocampus. Representative photomicrographs of TUNEL staining (a), active Bcl-2 immunohistochemistry (b), and active Bax immunohistochemistry (c). The cells with the brown-stained cytoplasm were the positive cells. Magnification: 400x. Scale bar = 20 *μ*m.

TUNEL assay was used to exam the typical DNA laddering pattern of neurons in the ischemic penumbra regions after cerebral I/R injury. As shown in Figure 6(a), we found that the number of apoptotic cells in the hippocampus region 24 h after BCCAO and reperfusion was increased significantly compared with the sham group. However, the number of apoptotic cells was decreased significantly in the 10 mg/kg NaB treatment group compared with model group, suggesting the antiapoptotic activity of NaB on the hippocampus.

### 3.6. NaB Activated BDNF-PI3K/Akt Pathway-Related Proteins (Caspase-3, Bcl-2, Bax, Akt, p-Akt, and BDNF) Assessed by Western Blot and Immunohistochemistry

**Figure 7:**
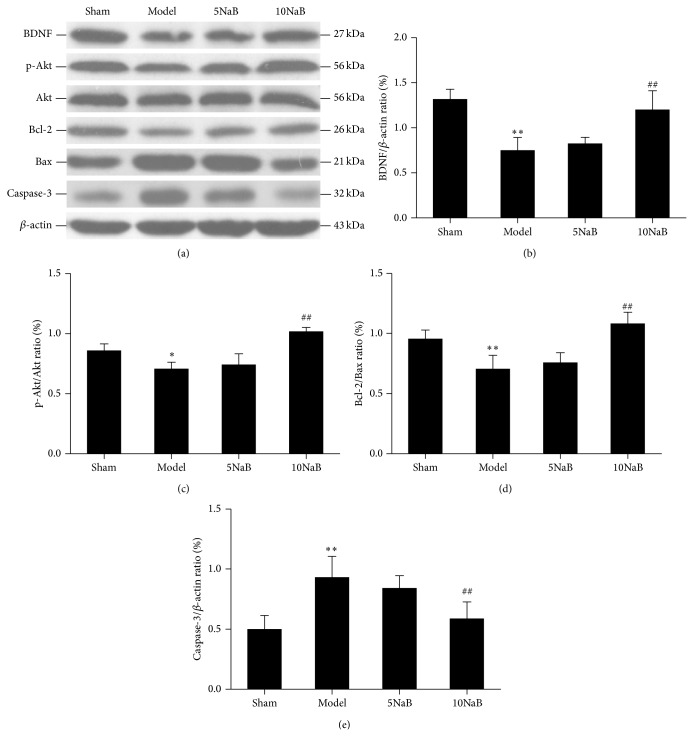
Effect of NaB on western blot of apoptosis-related protein (caspase-3, Bcl-2, Bax, Akt, p-Akt, and BDNF). Western blot studies of BDNF, p-Akt, Akt, Bcl-2, Bax, and caspase-3 (a) and the ratios of the protein levels of the BDNF/*β*-actin, p-Akt/Akt, Bcl-2/Bax, and caspase-3/*β*-actin (b), (c), (d), and (e). ^∗^ *p* < 0.05 versus sham group, ^∗∗^ *p* < 0.01 versus sham group, ^#^ *p* < 0.05 versus model group, and ^##^ *p* < 0.05 versus model group. *n* = 6, each group.

In order to evaluate the protective effect of NaB on BDNF-PI3K/Akt pathway after cerebral I/R injury, we examined the protein levels of caspase-3, Bcl-2, Bax, Akt, p-Akt (ser473), and BDNF by western blot analysis. The protein level of caspase-3 in the model group was abundantly increased compared with the sham group, whereas caspase-3 in 10 mg/kg NaB treatment group was dramatically decreased compared with the model group (Figures 7(a) and 7(e)). The 10 mg/kg NaB treatment markedly increased the levels of Bcl-2 and decreased the levels of Bax in mice after cerebral I/R injury (Figure 7(a)). The ratio of Bcl-2/Bax (antiapoptotic/proapoptotic) was significantly reduced in the model group compared with the sham group (*p* < 0.01, Figure 7(d)). However, the ratio of Bcl-2/Bax in 10 mg/kg NaB treatment group was remarkably increased compared with the model group (*p* < 0.01, Figure 7(d)). There was no significant difference in protein level of Akt among the four groups (*p* > 0.01, Figure 7(a)). However, the level of p-Akt in the model group was significantly reduced in comparison to the sham group, increased in 10 mg/kg NaB treatment group (Figure 7(a)). The ratio of p-Akt/Akt was significantly descended in the model group in comparison to the sham group (*p* < 0.05, Figure 7(c)). However, the ratio in 10 mg/kg NaB treatment group was significantly increased compared with the model group (*p* < 0.01, Figure 7(c)). The protein level of BDNF in the model group was significantly decreased compared with the sham group, whereas the 10 mg/kg NaB treatment group was significantly increased compared with the model group (*p* < 0.01, Figures 7(a) and 7(b)). There was no significant difference between the 5 mg/kg NaB treatment group and the model group (*p* > 0.05, Figure 7). These findings implied that NaB treatment could activate BDNF-PI3K/Akt pathway.

The above results were also partly confirmed by immunohistochemistry. As shown in Figures 6(b) and 6(c), few positive cells of Bax while plenty of positive cells of Bcl-2 could be found in the sham group. In the model group, the positive cells of Bax were numerous, while Bcl-2 was less compared with the sham group in the hippocampal CA1 region. After the NaB treatment, the number of the positive cells of Bax was significantly decreased, whereas the number of the positive cells of Bcl-2 was significantly increased in comparison to the model group.

## 4. Discussion

Although many mechanisms are involved in the pathogenesis of stoke, numerous studies show that oxidative stress, inflammation, and apoptosis mainly account for its pathogenic progression [22, 23]. The results of this study demonstrated that NaB may protect against cerebral I/R injury by improving neurological dysfunction and pathological changes, restoring the levels of MDA and SOD and decreasing the levels of proinflammatory cytokines IL-1*β*, IL-8, and TNF-*α* after cerebral I/R injury. The underlying mechanism of this antiapoptotic effect may be involved in the activation of the BNDF-PI3K/Akt pathway.

NaB, a dietary microbial fermentation product of fiber, possesses multiple bioactivities. Recent findings have shown that it could effectively inhibit inflammation and neuronal apoptosis in the animal of brain disorders [11]. Furthermore, it has markedly improved associative and spatial learning and memory in the mice of AD [24]. However, the protective role of NaB in cerebral I/R injury has not been clear. In order to develop cerebral I/R injury in mice, we well established cerebral I/R mice model induced by BCCAO [17, 18], which resulted in morphological and functional changes such as extensive death of hippocampal CA1 pyramidal neurons. The reports show a very wide range of effective doses of NaB [25–28], and these varieties in dosage might be related to the different animal species and models. According to the preliminary experiment, we chose the dosage of 5 and 10 mg/kg for NaB. Our results indicated that the NaB treatment effectively prevented neuropathological alterations in hippocampus thereby resulting in improvement of neurological deficit in mice after the cerebral I/R injury induced by BCCAO.

The brain is very sensitive to ROS-mediated injury. Numerous studies have shown that ROS are produced in the brain during I/R injury and directly involved in oxidative damage in ischemic tissue, which led to cell death [29]. SOD is an endogenous antioxidant, which plays a role in the prevention of oxidative injury. Enhancement of antioxidant activities in brain tissues is beneficial for alleviating neuronal damage from cerebral I/R [5, 30, 31]. Recent studies have indicated that NaB possesses an antioxidant feature in the intestinal epithelial cells [32]. However, its antioxidant effects on cerebral I/R have not yet been reported in mice. We found that 10 mg/kg NaB treatment markedly restored the levels of MDA and SOD after the cerebral I/R injury.

Inflammatory reaction plays a vital role in aggravating brain damage during acute stoke [6]. After the occlusion of cerebral blood flow, tissue damage was accompanied by the inflammatory reaction. Cytokines such as IL-1*β*, IL-8, and TNF-*α*, as mediators for regulating the innate and adaptive immune systems, are changed in the brain with various diseases including ischemic brain injury [2, 7]. In the present study, levels of IL-1*β*, IL-8, and TNF-*α* were markedly increased in the model group relative to the sham group. Consistently, this study found that 10 mg/kg NaB treatment alleviated cerebral I/R injury by lessening inflammatory factors IL-1*β*, IL-8, and TNF-*α*. The fact that NaB could provide anti-inflammatory effects in acute lung injury has been reported [15].

Oxidative stress and inflammation can also cause neuronal apoptosis. Apoptosis plays an important role in the process of neuronal death after cerebral ischemia [33, 34]. Recent studies have reported that NaB exerted neuroprotective effects by inhibiting neuronal apoptosis [31]. As a result of NaB treatment, a marked reduction in the number of TUNEL-positive cells in the hippocampus was observed.

It has been reported that BDNF could exert neuroprotective effect against ischemic brain injury in vivo via activating PI3K/Akt signaling pathway [35]. Akt, downstream of PI3K, is considered to be one of the important factors in cell growth and survival. It is well known that Bcl-2, Bax, and caspase-3 are the downstream target of Akt [36]. Akt activation suppresses apoptosis and promotes cell survival by phosphorylation and subsequent inactivation of apoptosis-inducing factors. Bcl-2 is an antiapoptotic factor and important for cell survival while Bax promotes apoptosis. Caspase-3, a key factor in apoptosis, eventually leads to DNA fragmentation and triggers apoptosis in the acute stage of stroke [23, 37]. Activation of PI3K/Akt pathway has been widely reported to participate in the protection against cerebral I/R injury [38]. In the present study, our result showed that 10 mg/kg NaB treatment significantly enhanced the phosphorylation of Akt. Simultaneously, 10 mg/kg NaB treatment markedly increased the levels of BDNF, p-Akt, and Bcl-2 and decreased the levels of Bax and caspases-3 in mice after cerebral I/R injury. Taken together, these results indicated that BDNF-PI3K/Akt pathway might be involved in antiapoptotic effect of NaB on cerebral I/R injury in mice.

## 5. Conclusion

In summary, this study firstly demonstrated that NaB attenuated cerebral I/R injury by minimizing oxidative stress and inflammatory and reducing apoptosis in vivo. Meanwhile, NaB exerted its antiapoptotic properties in cerebral I/R injury via BDNF-PI3K/Akt signaling pathway and showed a therapeutic potential for treatment of stoke in clinic.
